# Exome Sequencing in an ADSHE Family: VUS Identification and Limits

**DOI:** 10.3390/ijerph191912548

**Published:** 2022-10-01

**Authors:** Chiara Villa, Federica Arrigoni, Eleonora Rivellini, Marialuisa Lavitrano, Luca De Gioia, Luigi Ferini-Strambi, Romina Combi

**Affiliations:** 1 School of Medicine and Surgery, University of Milano-Bicocca, 20900 Monza, Italy; 2 Department of Biotechnology and Biosciences, University of Milano-Bicocca, 20126 Milan, Italy; 3 Department of Clinical Neurosciences, Neurology-Sleep Disorder Center, IRCCS San Raffaele Scientific Institute, 20127 Milan, Italy; 4 Department of Clinical Neurosciences, Vita-Salute San Raffaele University, 20127 Milan, Italy

**Keywords:** mutation, ADSHE, epilepsy, gene

## Abstract

Autosomal dominant sleep-related hypermotor epilepsy (ADSHE) is the familial form of a focal epilepsy characterized by hyperkinetic focal seizures, mainly arising during non-rapid eye movements (NREM) sleep. Mutations associated with ADSHE account for a small proportion of the genetically determined cases, suggesting the existence of other disease-causing genes. Here, we reported the results obtained by performing trio-based whole-exome sequencing (WES) in an Italian family showing ADSHE and investigated the structural impact of putative variants by in silico modeling analysis. We identified a p.(Trp276Gly) variant in *MOXD1* gene encoding the monooxigenase DBH like 1 protein, cosegregating with the disease and annotated as VUS under the ACMG recommendations. Structural bioinformatic analysis predicted a high destabilizing effect of this variant, due to the loss of important hydrophilic bonds and an expansion of cavity volume in the protein hydrophobic core. Although our data support a functional effect of the p.(Trp276Gly) variant, we highlight the need to identify additional families carrying *MOXD1* mutations or functional analyses in suitable models to clarify its role in ADSHE pathogenesis. Moreover, we discuss the importance of VUS reporting due to the low rate of pathogenic variant identification by NGS in epilepsy and for future reinterpretation studies.

## 1. Introduction

Autosomal dominant sleep-related hypermotor epilepsy (ADSHE), previously known as autosomal dominant nocturnal frontal lobe epilepsy (ADNFLE) [1], is the familial form of SHE, a rare focal epilepsy characterized by stereotyped hyperkinetic seizures, mostly arising during the non-rapid eye movement (NREM) stage of sleep. Seizures originate in the frontal lobes, as well as in other neocortex regions [2,3]. They are typically short (<2 min) in duration and occur frequently, usually during stage 2 NREM sleep. The confirmed diagnosis relies on a clinical history and video-electroencephalogram (EEG) documentation of hypermotor nocturnal motor manifestations [1,4]. SHE occurs in both genders at any age, but the majority of individuals develop the disease before 20 years of age with a predominance in childhood and adolescence. The disorder has probably been underestimated or in many cases misdiagnosed as parasomnias, mainly in children [5]. Low doses of carbamazepine in a single bedtime administration represents the first-line option drug in SHE patients and it is associated with remission in approximately 70% of individuals [6,7]. However, about one-third of patients are resistant to the drug, and surgery can be the only effective treatment in patients showing poor outcome after a long follow-up [5].

ADSHE follows an autosomal dominant pattern of inheritance with incomplete penetrance, estimated to 70% [8]. No clear genotype–phenotype correlation exists and symptoms may be very variable even among members of the same family [9]. ADSHE have been associated with mutations in genes encoding the α4, β2, and α2 subunits of the neuronal nicotinic acetylcholine receptors (*CHRNA4*, *CHRNB2,* and *CHRNA2*, respectively) [10], the corticotropin-releasing hormone (*CRH*) [11], the sodium-gated potassium channel (*KCNT1*) [12], and components of the mTORC1/GATOR1 complex (*DEPDC5*, *NPRL2,* and *NPRL3*) [13,14,15]. However, currently known mutations explain approximately 30% of the genetically determined cases [16], suggesting that additional causative genes exist. 

In this study, we report results obtained by performing trio-based whole exome sequencing (trio-WES) in family members affected by ADSHE, who do not carry mutations in already known genes, and we investigate the structural impact of putative variants by in silico modeling analysis. Moreover, we discuss the importance of reporting the identification of VUS due to the low rate of pathogenic variant identification using this strategy in epilepsy.

## 2. Materials and Methods

### 2.1. Sample Composition

**Figure 1:**
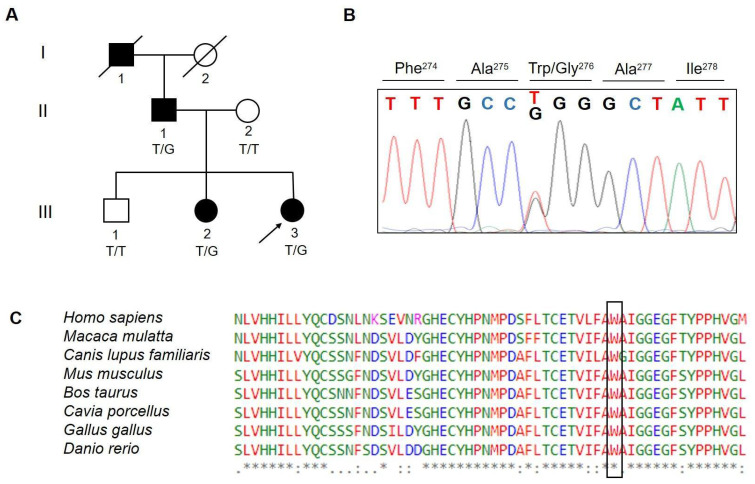
(**A**) Family pedigree of the proband showing the identified variant. The arrow points to the proband. Genotypes for available individuals are shown as T/T, wild-type (WT) genotype and T/G, heterozygous genotype; (**B**) sequencing chromatogram including the identified p.(Trp276Gly) variant is shown; (**C**) multiple alignments using ClustalW and amino acid conservation of MOXD1 p.(Trp276Gly) across several species. Letters in the box denote amino acids substituted. An asterisk denotes conserved amino acid.

The sample is composed of an Italian family showing four cases of SHE (Figure 1A) and with a typical autosomal dominant inheritance. Three affected individuals were still alive and participated in the study as well as two healthy members of the family. The study was approved by an institutional ethical committee (University of Milano-Bicocca, Milano, Italy). Written informed consent was obtained from all participants in the study. The study did not involve children/minors.

Since the age of 12 years, the family proband had recurrent episodes, characterized by a sudden elevation of head and trunk with fearful expression and pelvic thrusting. Episodes occurred 5 to 10 times per week, sometimes even in daytime. The main nocturnal clinical features were represented by shouting and moaning. She was treated with carbamazepine 400 mg once a day for two years with only partial efficiency. Then, she decided to stop therapy and she did not attend a follow-up. The proband’s sister reported the same type of episodes but with less frequency and never underwent pharmacological treatment. The father reported episodes in childhood and spontaneous remittance when he was 19 years old.

A video-polysomnography showed 10.3 attacks per hour. A sleep EEG showed diffuse background flattening, while an awake EEG was normal.

### 2.2. Whole Exome Sequencing and Data Processing

Genomic DNA (gDNA) of all the available family members was extracted from peripheral blood using a QIAamp^®^ Blood Mini Kit (QIAGEN, Hilden, Germany), according to the manufacturer’s instructions. Quality control was applied to DNA samples (≥500 ng needed per reaction at a concentration of ≥50 ng/µL in a final volume of ≥20 µL, A260/280 = 1.7–2, and integrity checked by agarose electrophoresis). To detect causal variants, whole-exome sequencing (WES) was performed on trio samples using standard Agilent (Santa Clara, CA, USA) and Illumina (San Diego, CA, USA) protocols. Briefly, libraries were constructed from 500 ng of gDNA and enriched with an Agilent SureSelect Human All Exon V7 Kit (Agilent Technologies, Santa Clara, CA, USA) to capture the consensus coding sequence (CCDS) of exonic and flanking intronic regions of approximately about 48 Mb. Generated libraries were sequenced using the Illumina HiSeq 2500 platform to obtain paired-end reads of 150 base pairs (bp) with an average coverage depth of approximately 100×. Typically, more than 92% of the targeted region was covered over >20×, with a read enrichment greater than 76%.

Bases and reads with a quality score below 30 were trimmed off. Then, the clear reads were mapped against the human genome reference assembly (UCSC Genome Browser hg19) with a Burrows-Wheeler Aligner (BWA, version 0.7.7) [17], resulting in SAM/BAM output. Polymerase chain reaction (PCR) duplicates were then flagged with Picard. Single nucleotide variations (SNVs) and insertions/deletions (indels) were detected using SAMtools (version 0.1.19) [18]. The alignment strategy produced a percentage of aligned reads of 99.8% and a percentage of aligned bases of 96%. Realignment of small indels, base calibration, variant calling, and filtering were then performed using a Genome Analysis Tool Kit (GATK, version 4.1.6.1). Individual and trio analyses were both performed. In the latter case, a joint variant calling strategy was applied. Sequence variants were further annotated with ANNOVAR using population and literature databases, including the Genome Aggregation Database (gnomAD), the 1000 Genomes Project (1000G), the Exome Aggregation Consortium (ExAC), Clinvar, HGMD, and OMIM. The possible pathogenicity of variants was predicted according to the online tools SIFT (https://sift.jcvi.org/ (accessed on 1 February 2022)), PolyPhen2 (https://genetics.bwh.harvard.edu/pph2/ (accessed on 1 February 2022)), MutationTaster (https://www.mutationtaster.org/ (accessed on 1 February 2022)), and CADD score (http://cadd.gs.washington.edu/snv (accessed on 1 February 2022)). 

WES and data processing were both carried out at Biodiversa (Treviso, Italy) as a service.

### 2.3. Variant Confirmation and Familial Segregation Analysis

Sanger sequencing, in forward and reverse directions, was performed on all the available family members to validate candidate variants and to evaluate segregation with the phenotype in this pedigree. Primers amplifying the target regions were designed using the web-based Primer3.0 (http://bioinfo.ut.ee/primer3-0.4.0 (accessed on 1 February 2022)) server and their specificity was checked by Primer blast on the NCBI genome browser (https://www.ncbi.nlm.nih.gov/tools/primer-blast/). PCRs were performed directly on 50–100 ng of genomic DNA in standard conditions, using the following primers: 5′-TGGGATGTTGTAGGGTGGTC-3′ and 5′-TCCCTTCCTACGAAAATGGAGT-3′ for *MOXD1* and 5′-ACAAGGTTGAGTGCGGAGAG-3′ and 5′-ACAATGGTCTCACACTCCCA-3′ for *SYNE2*. Sequencing was carried out directly on both strands of purified PCR products by using the BigDye Terminator Cycle Sequencing kit v3.1 and an automated ABI-3130 DNA sequencer (Applied Biosystems, Foster City, CA, USA). ChromasPro v1.34 (Technelysium Pty Ltd., South Brisbane, Australia) software was used for mutation detection.

### 2.4. Computational Analysis of MOXD1 and Mutant Structural Features

The MOXD1 ab initio model was retrieved from the AlphaFold Protein Structure Database (AlphaFold DB, https://alphafold.ebi.ac.uk (accessed on 1 April 2022)) [19]. The change in protein stability (ΔΔG, kcal/mol), upon site-specific mutations was calculated using multiple tools, namely DynaMut2 [20], SDM [21], mCSM [22], DUET [23] and DeepDDG [24]. The predicted ΔΔG values represent the difference in the ΔG of protein folding between wild type and mutants, calculated as ΔΔG = ΔG(mutant) – ΔG(wild type), therefore, negative values of ΔΔG were associated with protein destabilization upon the considered mutation. The Missense 3D tool [25] was used to detect structural damages associated with a mutation. A detailed structural inspection of wild-type and mutated forms was carried out by generating 3D models for the mutant, starting from the MOXD1 AlphaFold model. The latter was first prepared using the Schrodinger’s Protein Preparation Wizard [26,27], in order to add missing H atoms, possible disulphide bonds, and optimal formal oxidation/protonation states (at pH 7). This procedure also involved optimizing H-bonds and performing a structural relaxation that freely minimized H atoms and achieved restrained optimization (0.3 Å RMSD) of heavy atom positions, in order to relax introduced steric strains, if any. Then, in silico mutagenesis was carried out with the BioLuminate modeling platform [28]. The mutant model was then refined with a local minimization tool (with a cutoff of 5 Å around the mutated site). Then, BioLuminate was also used to detect hydrogen bonds and hydrophobic interactions of both wild-type and mutated side-chains with proximal residues. The cDART database [29] was used to define domain composition and ordering in MOXD1. PyMOL was used to generate figures [30].

## 3. Results

### 3.1. Variant Filtration and Prioritization

WES was performed in three individuals belonging to the family: the proband (III-3) and her parents (II-1 and II-2) (Figure 1A). 

Since we assumed the autosomal dominant pattern of inheritance, we searched for rare heterozygous variants shared among affected members but presented as a homozygous reference genotype in the healthy parent. 

After annotation, a merged file containing only variants common to the proband and affected father but absent in the healthy mother was generated using a joint variant calling pipeline. Variants with low quality score and depth of coverage were removed. To narrow down the number of candidate variants, a filtration strategy was applied using the following rules: (1) For mode of inheritance, heterozygous variants were of interest, given the rarity of disease transmitted with an autosomal dominant pattern; (2) for population frequency, variants with minor allele frequency (MAF) < 0.01% in population-level allele frequency data derived from the gnomAD (gnomad.broadinstitute.org), ExAC (exac.broadinstitute.org), and 1000GP (internationalgenome.org) were selected; (3) for location-based filtering, intronic and intergenic variants were removed; (4) for variant effect, only variants that were non-synonymous, frameshift, and nonsense, or affected canonical splice-site donor/acceptor sites were retained; (5) finally, for predicted impact, the potential impacts of given variants were assessed for being disease-causing by SIFT, PolyPhen2, MutationTaster, and CADD.

By filtering out variants, three heterozygous missense variants (Table 1) were identified. Using PCR-based Sanger sequencing, the heterozygosity of identified variants was validated in the proband and affected father and their absence in the healthy mother was confirmed.

Among them, a novel variant was identified in the *ASCL2* (achaete–scute complex homolog 2) gene. We classified the variant as a VUS considering PM2 (absent in ExAc, gnomAD, and 1000GP) and PP3 (in silico). The gene, already associated with the Beckwith–Wiedemann (BWS) syndrome, is a maternally expressed imprinted gene particularly important during implantation of the developing embryo, specifically in placental development and neuronal precursor determination. Due to its maternal expression, we considered that it was probably not directly involved in the ADSHE phenotype, which, in our family, was inherited from the affected father. 

Sanger sequencing of the other affected (III-2) and unaffected (III-1) family compliant members was performed to investigate the remaining two variants’ segregations with the disease within the family. This excluded co-segregation between the *SYNE2* (spectrin repeat containing nuclear envelope protein 2) variant and the disease, in fact, the variant was not detected in the affected sister of the proband (III-2) (Figure 1A). 

The *MOXD1* variant was also found in the heterozygous state in the affected sister (III-2) of the proband, while it was absent in the healthy brother (III-1), thus, co-segregating with the disease (Figure 1A,B). 

The amino acid change in *MOXD1* was predicted to be pathogenic and affected a highly evolutionary conserved amino acid (Figure 1C). We classified this variant under the ACMG criteria as a VUS considering PM2 (absent in ExAc and 1000GP), moderate evidence of co-segregation (PP1 > M), and PP3 (in silico).

### 3.2. Modeling

**Figure 2:**
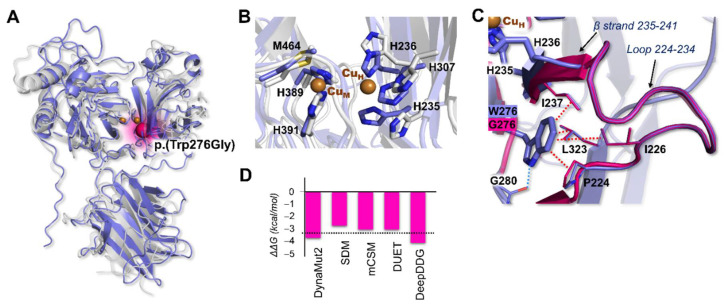
(**A**) 3D model of MOXD1 (in pale blue) superimposed on the crystal structure of hDBH monomer (in grey, PDB ID: 4ZEL). The N- and C-terminal portions (1–22 and 582–613, respectively) of the MOXD1 model were not considered, since these regions of the AlphaFold model were characterized by low (70 < pLDDT < 50) or very low (pLDDT < 50) confidence. The positions of the two copper ions, absent in the predicted model, were modeled to complete the overall architecture of each Type II Cu site. The p.(Trp276Gly) mutation was mapped onto the 3D model as pink spheres; (**B**) Cu_H_ and Cu_M_ sites in MOXD1 (pale blue) and DBH (in grey); (**C**) superimposition of wild-type MOXD1 (in pale blue) and of the p.(Trp276Gly) mutant (in pink) models. In the wild-type, the hydrogen bond between G280 and W276 is highlighted as a dotted light blue line, while hydrophobic contacts with P224, I226, I237, and L323 are shown as dotted red lines; (**D**) predicted ΔΔG values. The average value obtained for each mutation is indicated by a horizontal black dotted line.

The 3D model of the monooxygenase DBH-like protein 1 (MOXD1) deposited in AlphaFold DB was compared to the structure of the hDBH monomer (PDB ID: 4ZEL) [31], the copper monooxygenase sharing the highest sequence identity (32%). The two proteins present the same domain compositions, according to the cDART database (Figure S1), in particular: a DOMON-like domain, containing (in contrast to DBH) a conventional heme-binding pocket [32], and two Type II copper-monooxygenase domains [33]. Structural alignment of the two proteins was used to identify the putative bimetallic MOXD1 active site, which was identical to that of DBH (Figure 2A,B), formed by the two monooxygenase domains, each participating with a typical Type II copper center. One Cu center, labeled Cu_H_ (belonging to the Cu_H_ domain), is coordinated by residues H235, H236, and H307 (H262, H263, and H333 in DBH) while the second, Cu_M_ (belonging to the Cu_M_ domain), features a first coordination sphere formed by H389, H391 and M464 (H412, H414, and M487 in DBH).

W276 is located in a β strand close to the Cu_H_ binding site (Figure 2A,C), at around 5 Å from the H236 alpha carbon. In the wild type, W276 establishes one hydrogen bond with G280 and multiple hydrophobic contacts with amino acids belonging to the loop 224–234 (I226, P224, L323) and to the β strand 235–241 (I237), which bears both H235 and H236 that take part in Cu_H_ coordination. These interactions keep the protein structure locally stable and cohesive. Since W276 is in high proximity to the Cu_H_ coordination neighborhood, a mutation that causes the total loss of such interactions (e.g., p.(Trp276Gly)) may reasonably affect the integrity/shape of the active site and/or alter its catalytic activity. This observation was qualitatively confirmed by using a series of well-established computational tools to predict the impact of a specific mutation on protein stability (Figure 2D). Indeed, a consensus result was obtained with all the tested codes that predicted p.(Trp276Gly) as highly destabilizing (ΔΔG range, −4.2–−3.8 kcal/mol and average ΔΔG, −3.4 kcal/mol). In addition, the Missense 3D tool detected structural damage upon mutation to G, caused by an expansion of cavity volume in the protein hydrophobic core by 147.96 Å^3^.

## 4. Discussion

In this paper, we report the results of exome sequencing and reveal a variant p.(Trp276Gly) within the *MOXD1* gene co-segregating with ADSHE in an Italian family. The variant was found in heterozygosis in all available individuals affected by ADSHE and it was inherited from the affected father. The variant changed the highly conserved tryptophan to a glycine located closed to the Cu_H_ binding site which is encoded by the exon 5 of the gene. We classified the variant as VUS under the ACMG recommendation considering PM2 (absent in ExAc and 1000GP), moderate evidence of co-segregation (PP1 > M), and PP3. In fact, a deep in silico modeling analysis of the variant’s effects showed that the amino acid change provoked the absence of hydrophilic bonds that are important for protein stability and structural damage caused by an expansion of cavity volume in the protein hydrophobic core.

*MOXD1* encodes the monooxygenase DBH-like protein 1, also referred to as monooxygenase X (unknown substrate) or dopamine b-hydroxylase related. It is a member of the copper monooxygenase family, including dopamine monooxygenase (dopamine β-monooxygenase, DBM) and peptidylglycine α-hydroxylating monooxygenase (PHM), located on chromosome 6 [34]. MOXD1 expression was detected at the boundary of the neural plate (stage 7) during the development of the neural crest, and was distributed along in several brain areas: cerebellum, olfactory bulb, brain stem, and parietal cortex. Knockdown experiments demonstrated the role of MOXD1 in regulating the ER stress–mitochondrial apoptosis pathway, suggesting that it may maintain ER homeostasis under normal circumstances [35].

To date, *MOXD1* has not been directly associated with epilepsy, thus, the existence of a role in determining a phenotype such as ADSHE could not be inferred. However, differences in dopamine β-hydroxylase immunoreactivity have been reported between the brains of genetically epilepsy-prone and Sprague-Dawley rats [36]. 

Moreover, it has been reported that dopamine-β-hydroxylase knock-out (DBH KO) mice demonstrated reduced latencies to a first behavioral sign of seizure [37]. In particular, DBH KO mice were much more seizure sensitive than neuropeptide Y (NPY) KO.

A relationship between *MOXD1* and sleep regulation has been suggested by two studies. In the first study, aimed at determining the network of genes regulated by CLOCK (a regulator of circadian rhythms important in setting the sleep timing and stress responses), the authors identified groups of highly coexpressed genes. Among them, the CM3 was a module enriched in genes involved in neuronal development and projection assembly in addition to genes implicated in several neuropsychiatric disorders, as mentioned above, such as ASD, intellectual disability, and epilepsy. *MOXD1* was included in this module and was upregulated [38]. The second study demonstrated the expression of MOXD1 protein in the preoptic area (POA) of the brain, a region that contains a diverse group of neurons that differentially control wake, NREM sleep, and REM sleep. *Moxd1* expression can be modified by hormonal influences during the perinatal period, but not in adulthood [39].

Interestingly, by in situ hybridization, it was demonstrated that the expression pattern of *Moxd1* changed at different stages of development: *Moxd1* mRNA was first detected at P3; it further increased at P8, and remained similar at P21, whereas it appeared that fewer cells were labeled in the adult subplate [40]. 

The importance of stage-dependent expression of MOXD1 has been recently confirmed by two studies that identified novel regulators of neuronal differentiation and neurogenesis, both acting on MOXD1 expression. In the first study, Douka et al. identified a lncRNA (LINC01116) moderately expressed in the developing human forebrain and highly expressed in the developing human midbrain and spinal cord [41]. The LINC01116 peptide exhibited a cytoplasmic distribution and was detected in neurites and its siRNA knockdown, upon differentiation, resulted in a reduction in MOXD1 expression levels, thus, impeding neurite outgrowth. In the second study, the authors identified a novel species-specific regulator of early human neurogenesis, i.e., the miR-934 displayed a developmental stage-specific expression pattern during neural induction that was characterized by neural progenitor expansion and early neuron generation. By RNA profiling of hESC-derived cells at the neural induction phase upon sustained miR-934 inhibition, the authors demonstrated altered expression of genes including *MOXD1* [41]. MOXD1 was also reported as a signature of parvalbumin interneurons and it has been suggested that it could contribute to the local inactivation of neurotransmitter in striatal dopaminergic synapses, although this function has not been demonstrated [42].

Little is known about the networks and mechanisms involving MOXD1 in brain development and process regulation. This lack of knowledge strengthens the observation that, although a functional effect of the mutation was demonstrated by our results in silico, a direct role of the p.(Trp276Gly) in ADSHE pathogenesis has still to be proven. This could be done only by the identification of new affected families with a mutation in *MOXD1* co-segregating with the disease or by the development and study of specific transgenic mouse models. However, we think that it could be very important for future analysis to report VUS in the literature. The classification of a variant as a VUS, in fact, means that, at the moment of its detection, there were insufficient data to determine if the variant is really related to the disease. The ACMG, in these cases, recommends pursuing follow-up testing aimed at generating new evidence for a reclassification of each single variant. As already stated in our case, this would require identifying additional families or new functional studies performed by other groups of researchers. Then, transparency in reporting could be achieved, which is extremely important in reclassification.

This is of particular importance when studying the genetics of epilepsy. In fact, these studies are complex due to the high genetic heterogeneity and the frequently reported low penetrance of mutations detected even in monogenic forms. Several genetic tests based on NGS techniques are already available ranging from targeted resequencing of panels of genes known to be associated with epilepsy to exome sequencing and even also genome sequencing. The study of familial clusters instead of single sporadic patients is important to correctly choose the parameters needed to filter variants. In our family, for example, we were able to select only rare heterozygous variants that were detected only in all affected family members, due to the observation that the pedigree followed a classical autosomal dominant inheritance pattern and to the knowledge on the rarity of the disease (and thus, of the involved variants). 

The identification of a VUS is challenging for researchers and for clinicians, because its role continues to be uncertain and it is difficult to make it understandable to patients. In our case, we identified a variant with very low frequency in a population, predicted to damage and alter the functionality of the relevant protein, cosegregating with the disease in the family. However, the importance of this detection for the patient is limited by our partial knowledge: the gene has only been studied in a limited number of papers in the literature; the real activity of the protein is only partially known; there are no data on the enzyme substrate, thus, the possibilities of functional analyses in vitro and in vivo are limited; the variant could be involved in other phenotypes affecting the family and not reported yet to the clinicians. These are the reasons why clinicians have a crucial role in detailed history taking and in setting out accurate phenotypic characterization. 

In the upcoming era of precision medicine aimed at establishing aetiology-based treatment and management of each single patient, the transparency of all genetic aetiological data obtained by NGS is crucial.

In case new studies should confirm the involvement of *MOXD1* in ADSHE, the stage-dependent expression of this gene could be an important aspect to take into consideration when deciding therapy for a precision medicine approach. In fact, according to the observations in our patients, the epileptic phenotype could disappear or at least diminish in adulthood even without drug treatment. Then, it would be important to evaluate the severity and frequency of seizures and set up drug therapy only when essential.

## 5. Conclusions

In summary, we conducted a trio-based WES study in an Italian family affected by ADSHE, followed by variant prioritization strategies. Our analysis led to the identification of a rare heterozygous VUS in the *MOXD1* gene (p.(Trp276Gly)) co-segregating with the clinical phenotype. In silico modeling analysis predicted a significant loss in protein stability for this variant, likely impairing both protein functionality and integrity. Nevertheless, further investigations based on the identification of other families harboring *MOXD1* mutations and functional studies are needed to highlight the relevance of this gene in the pathogenesis of ADSHE and to clearly classify VUS.

## Figures and Tables

**Table 1:** Rare variants identified by exome sequencing and variant prioritization.

Chr	Position (GRCh37)	Ref	Alt	Gene ID	Effect	Feature_ID:HGVS.c:HGVS.p	CADD PHRED Score
Chr6	132649571	A	C	*MOXD1*	Nonsynonymous SNV	MOXD1:NM_015529:exon5: c.T826G:p.Trp276Gly	29.9
Chr11	2290998	A	T	*ASCL2*	Nonsynonymous SNV	ASCL2:NM_005170:exon1: c.T565A:p.Trp189Arg	33.0
Chr14	64655368	A	C	*SYNE2*	Nonsynonymous SNV	SYNE2:NM_015180:exon98: c.A17813C:p.Asn5938Thr	25.5

Chr, chromosome; Ref, reference allele; Alt, altered allele; HGVS, human genome variation society.

## Data Availability

The data will be available from the corresponding authors upon reasonable request.

## Supplementary Materials

The following supporting information can be downloaded at: https://www.mdpi.com/article/10.3390/ijerph191912548/s1, Figure S1: 3D model of MOXD1 and cDART comparison to DBH.
