# Supplementary material for: Exome Sequencing in an ADSHE Family: VUS Identification and Limits
Source: Int J Environ Res Public Health. 2022 Oct 1;19(19):12548. doi: 10.3390/ijerph191912548 (PMC9565017; doi:10.3390/ijerph191912548)
Supplement: Supplementary file 1 [file ijerph-19-12548-s001.zip › ijerph-1933317-supplementary.pdf]

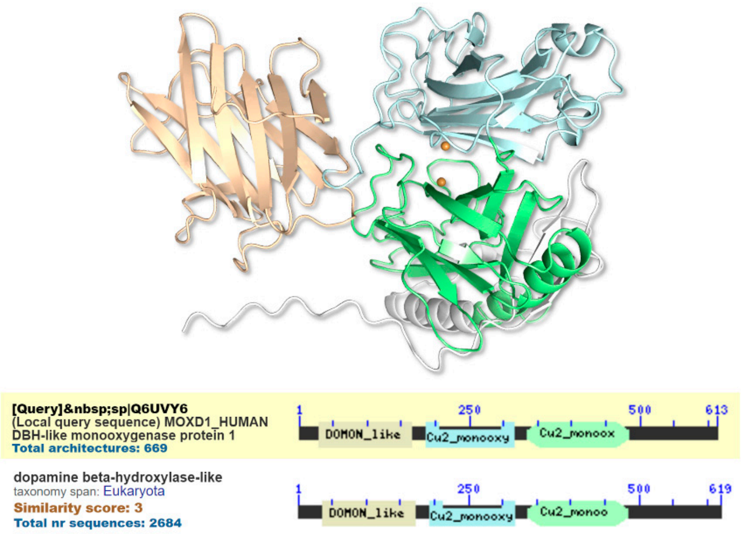

Figure S1. 3D model of MOXD1 and cDART comparison to DBH. Top: 3D model of MOXD1 with the three domains put in evidence (DOMON-like in sand, Cu_H in cyan and Cu_M in green); bottom: cDART output for MOXD1 in comparison to DBH.
